# Supplementary material for: The Protein Landscape of Mucinous Ovarian Cancer: Towards a Theranostic
Source: Cancers (Basel). 2021 Nov 9;13(22):5596. doi: 10.3390/cancers13225596 (PMC8616050; doi:10.3390/cancers13225596)
Supplement: Supplementary file 1 [file cancers-13-05596-s001.zip › Supplementary File S1 - Search Strategy[1].pdf]

Supplementary File S1: Search Strategy

1. Key words

Population	Exposure
Mucinous ovarian cancer<br>Mucinous ovarian carcinoma<br>Epithelial ovarian cancer<br>Epithelial ovarian carcinoma<br>Type I-II tumours<br>Ovarian carcinoma<br>Ovarian neoplasm	Proteomics<br>Immunohistochemistry<br>Targeted therapy<br>Molecular therapy

2. Ovid MEDLINE (2010-2021)

1. exp Proteomics/
2. proteomic*.mp.
3. exp Immunohistochemistry/
4. immunohistochemistry.mp.
5. theranostic*.mp.
6. 1 OR 2 OR 3 OR 4 OR 5
7. mucinous ovar*.mp.
8. 6 AND 7

3. PUBMED (2010-2021)

1. “Proteomics”[Mesh]
2. proteomic*
3. “Immunohistochemistry”[Mesh]
4. immunohistochemistry
5. theranostic*
6. 1 OR 2 OR 3 OR 4 OR 5
7. “mucinous ovar*”
8. 6 AND 7

4. Google Scholar (2010-2020)
Search: ((proteomics) OR (theranostic) OR (theranostics)) AND ((“mucinous ovarian cancer”) OR (“mucinous ovarian carcinoma”)

5. Ovid MEDLINE (2010-2021)

1. exp Ovarian Neoplasms/
2. “mucinous*”.af.
3. Adenocarcinoma, Mucinous/
4. “ovar*”.af.
5. (mucinous* adj4 ovar*).af.
6. 1 AND 2
7. 3 AND 4
8. 5 OR 6 OR 7
9. exp Proteomics/
10. exp mass spectrometry/
11. “spectrometr*”.af.

12. “proteo*”.af.
13. 9 OR 10 OR 11 OR 12
14. 8 AND 13

6. PUBMED (2010-2021)

1. “Ovarian Neoplasms”[Mesh]
2. Mucinous*
3. “Adenocarcinoma, Mucinous”[Mesh]
4. Ovar*
5. 1 AND 2
6. 3 AND 4
7. 5 OR 6
8. “Proteomics”[Mesh]
9. “Mass Spectrometry”[Mesh]
10. Spectrometr*
11. Proteo*
12. 8 OR 9 OR 10 OR 11
13. 7 and 12

7. Inclusion and exclusion criteria

Immunohistochemistry

Inclusion criteria	Exclusion criteria
Studies that assessed protein expression profiles via immunohistochemistry in MOC tissue samples	Serum analysis only<br>Immunohistochemistry was not used to quantify protein expression profiles<br>Did not present data on immunohistochemistry findings specific to primary MOC<br>Sample size ≤ 5, unless supported by other studies<br>Experimental analyses were not performed<br>Language other than English<br>Abstract or full text was not available<br>Published prior to the year 2010

Proteomics

Inclusion criteria	Exclusion criteria
Studies that investigated aberrant protein expression by means of proteomic analysis.	Serum analysis only<br>Proteomics was not used to quantify protein expression profiles<br>Did not present data on proteomic findings specific to primary MOC<br>Experimental analyses were not performed<br>Language other than English<br>Abstract or full text was not available<br>Published prior to the year 2010

An additional search was conducted using:

(("ovarian neoplasms"[MeSH Terms] OR ("ovarian"[All Fields] AND "neoplasms"[All Fields]) OR "ovarian neoplasms"[All Fields] OR ("ovarian"[All Fields] AND "cancer"[All Fields]) OR "ovarian cancer"[All Fields]) AND ("mucine"[All Fields] OR "mucines"[All Fields] OR "mucinous"[All Fields] OR "mucins"[MeSH Terms] OR "mucins"[All Fields] OR "mucin"[All Fields]) AND ("immunohistochemistries"[All Fields] OR "immunohistochemistry"[MeSH Terms] OR "immunohistochemistry"[All Fields])) AND (2010:2021[pdat])

And was filtered to obtain further detail on the proteins in Table 1.
