# Supplementary material for: The Protein Landscape of Mucinous Ovarian Cancer: Towards a Theranostic
Source: Cancers (Basel). 2021 Nov 9;13(22):5596. doi: 10.3390/cancers13225596 (PMC8616050; doi:10.3390/cancers13225596)
Supplement: Supplementary file 1 [file cancers-13-05596-s001.zip › Supplementary File S2 - Study Characteristics[1].pdf]

Supplementary File S2: STUDY CHARACTERISTICS

Table 1 Characteristics of immunohistochemistry studies in mucinous ovarian tumours (MOT) – Search 1

First Author	Year	DOI	Total MOT Cases	MOT by Subtype	Protein of Interest	Role of Target	Control Tissue Expression	Findings	Notes
Abdelaal, S	2016	10.14456/apjcp.2016.91/APJCP.2016.17.7.3295	20	Cystadenoma: 10<br>Borderline: 4<br>Carcinoma: 6	MDM2	Cell cycle regulator; negatively regulating p53.	Not reported	Cystadenoma: 0/10<br>Borderline: 0/4<br>Carcinoma: 5/6 cases positive	IHC Score was based on percentage of positive tumour cells being evaluated multiplied by intensity of tumour staining.<br><br>0 (negative): 0-5% positivity<br>1: 6-49% positivity<br>2: 50-100% positivity<br><br>0: no detectable immunostaining<br>1: weak or faint cytoplasmic and/or nuclear immunostaining<br>2: moderate<br>3: strong
Amsterdam, A	2011	10.3892/ijo.2011.1090	15-21*	Cystadenoma: 5-7<br>Borderline: 5-7<br>Carcinoma: 5-7	pERK1/2	Key role in induction of proliferation, differentiation and apoptosis.	Normal ovarian stromal tissue: 28.3±11.3% of nuclei (n=5).	Cystadenoma: 17.0±11.1% (p=0.24 against normal tissue)<br>Borderline: 68±9%<br>Carcinoma: 62.5±9.5%	Six to twelve areas of stained slides of each tissue sample was assessed.<br>Score: mean percentage of labelled nuclei in each photograph.<br><br>NOTE: authors do not state exactly how many cases per subtype of mucinous ovarian cancer.
Anglesio, M	2013	10.1002/path.4088	330	Borderline: 176<br>Carcinoma: 154	HER-2	Known oncogene	Not reported	MOC, 29/154 (18.8%) HER2-positive,<br>MBT, 11/176 (6.25%) HER2 positive (*p* = 0.0076).	HER2 was evaluated using an anti-HER2 antibody and scored visually according to the ASCO/CAP guidelines.
Armes, J	2013	10.1097/PAT.0b013e32835bd561	48	Cystadenoma: 15<br>Borderline: 16<br>Carcinoma: 17	AGR2	Unclear role of AGR2 in carcinogenesis; implicated in tumour cell growth, survival and metastasis	Normal ovarian epithelial tissue 11/36 (31%)	Cystadenoma: 15/15<br>Borderline MOC: 16/16<br>MOC: 17/17	Correlated expression of AGR2 with other tumour biomarkers: p53, p16, ER, CA125<br><br>NOTE: specific IHC scoring system detailed in previous reports.
Alshenawy, H	2010	10.1016/j.anndiagpath.2010.05.005	21	Carcinoma: 21	EGFR		Not reported	MOC:10/21	Positive if >10% of cells staining at any intensity
Ates, D	2016	10.1007/s12253-016-0040-2	22	Borderline: 15<br>Carcinoma: 7	PAX2<br>PAX8<br>CDX2	PAX2/8: association with pathogenesis uncertain.	Not reported	Borderline<br>PAX2: 1 -5: 12/15; 6-10: 3/15<br>PAX8: 1-5: 10/15; 6-10: 5/15; 11-15: 1/15<br>CDX2: 1-5: 6/15; 6-10: 5/15; 11-15: 4/15	IHC Scoring: Staining intensity (1-3); Staining distribution (1:<5%,2:5–25 %, 3: 25–50 %, 4: 50–75 %, 5: 75–100 %));<br>Multiplication of those two scores for score out of 15. 1-5 considered negative although this isn't clear.<br>PAX2 and PAX8 mostly negative although heterogeneity noted

								Carcinoma -<br>PAX: 21-5: 6/7; 6-10: 0; 11-15: 1/7<br>PAX8: 1-5: 2/7; 6-10: 4/7; 11-15: 1/7<br>CDX2: 1-5: 4/7; 6-10: 1/7; 11-15: 2/7	
Bassiouny, D	2018	10.1177/1066896917752861	36	Carcinoma: 36	CK7<br>CK20<br>CDX2<br>PAX8<br>HNFIB<br>AMACR<br>P53<br>HER2<br>WT1<br>SATB2<br>Chromogranin<br>Synaptophysin<br>ARID1A<br>MMR proteins	Various	Not reported	CK7: 31/36 – diffusely positive<br>CK20: 17/36 – diffusely positive; 4/36 focally positive<br>CDX2: 17/36 – diffusely positive; 2/36 focally positive<br>PAX8: 4/36 diffusely positive; 2/36 focally positive<br>HNFIB: 33/36 diffusely positive<br>AMACR: 29/36 diffusely positive; 4/36 focally positive<br>HER2: 6/35 positive<br>P53: 10/36 aberrant expression<br>MMR and ARID1A: all retained<br>PTEN: 4 lost expression<br>Chromogranin & Synaptophysin 5/36 positive<br>SATB2: 3/32 diffuse positive<br>P15 15/36 focal positive<br>Napsin A, ER, WT1: all negative, PR: 1/36 positive	p53 – null and overexpression (>75%) called aberrant<br>HER2 – as per guidelines<br>p16, >75% strong nuclear staining positive, otherwise negative<br>PTEN >1% positive, heterogenous If distinct areas <1%, >1%<br>MMR, ARID1A – absent or intact nuclear staining<br>Remainder: Percentage (score: 0 = less than 1%; 1 = 1% to 25%; 2 = 26% to 50%; 3 = 51% to 75%; 4 = 75% to 100%) Average across cores: Negative (score 0-1.5), focal positive (score 1.6-2.4), and diffuse positive (score 2.5-4). Intensity (score: 1-3). Average: negative (score 0), weak (0.1-1.5), moderate (1.6-2.4), and strong (2.5-3).
Chapel, D.B.	2020	10.1038/s41379-020-0642-9	10	Borderline: 5<br>Carcinoma: 5	INI<br>MTAP<br>p53<br>HER2	Various	Not reported	INI: retained in all cases<br>MTAP: lost in 1 borderline and carcinoma<br>p53: aberrant in 2 carcinomas<br>HER2: 3+ in 2 carcinomas; 2+ in one borderline	p53 – null and overexpression (>80%) called aberrant<br>INI and MTAP – complete absence in tumour cells<br>HER2 as per guidelines
Chen, S	2015	10.1186/s12885-015-1435-2	26	Carcinoma: 26	REG4	Protein activator of EGFR/Akt/AP-1 signalling pathway, through phosphorylation	Normal ovarian tissue – negative 20/26	REG4:<br>+: 6/26<br>++: 3/26<br>+++: 10/26	Positive expression was graded as follows: 0=negative; 1=1–49%; 2=50–74%; 3≥75%. Staining intensity : 1 = weak; 2 = intermediate; 3 = strong. Multiplied to obtain a final expression score as follows: −=0; +=1–2; ++=3–4; +++=6–9.
Cirstea, AE	2018	10.12865/CHSJ.44.02.06	13	Carcinoma:7<br>Borderline: 6	EGFR		Not reported	MOC 3/7<br>Borderline 1/7	Positive, any expression in >5% of cells
El-Balat, A	2018	10.1007/s12253-017-0240-4	44	Borderline	TFF3	Proliferation, migration, angiogenesis	Not reported	CIS < 1 – 38.6% (n=17)<br>CIS > 1 – 61.4% (n=27)	Combined intensity Score = SI x PP/1000 (with PP as percentage of stained cells; SI as staining intensity)
El-Gendi, S	2016	10.1007/s13277-015-4129-0	23	Benign: 4<br>Borderline: 7<br>Mucinous: 12	FGF18	Modulates tumour microenvironment by promoting neovascularisation	Not reported	Cystadenoma: 0 (0,0)<br>Borderline: 4 (4,6)<br>Carcinoma: 9 (8,9)	Score = percentage and intensity of positive cytoplasmic staining in tumour cells. Only reports strength in terms of a score – no specific mention of expression levels.<br>Expression increases with malignancy.
Engqvist, H	2019	10.1186/s12885-019-6084-4	29	Carcinoma: 29	COL3A1<br>GPR158	COL3A1 – may be associated with drug-resistance	Not reported	COL3A1: 29/29 (100%)<br>GPR158: 17/29 (58%) Mostly weak	Immunoreactive score (H-score) = based on percentage and intensity of positively stained cells

						GPR158 – unknown; similar proteins associated with tumorigenesis			Only discusses number of cases positive for significant protein expression, which was >0 for GPR158 and >20 for COL3A1<br>Also did RTKN2 and TTK but did not report results as these were not associated with survival.
Engqvist, H	2020	10.3389/fonc.2020.00162	29	Stage I - 22<br>Stage II - 7	CHEK1<br>FOXM1<br>KIF23<br>PARPBP	CHEK1 - DNA damage checkpoint<br>FOXM1 - cell proliferation<br>KIF23 - cell proliferation and migration<br>PARPBP - negative regulator of homologous recombination	Not reported	CHEK1: 83%<br>FOXM1: 86%<br>KIF23: 7%<br>PARPBP: 41%	H-score = based on percentage and intensity of positively stained cells (out of 300) . Threshold for positivity determined by association with outcome in Xtile: CHEK1 (>17), FOXM1 (>55), KIF23 (>40), PARPBP (>167)<br>Staining pattern mostly homogenous. However, CHEK1 and PARPBP demonstrated partially non-homogenous staining pattern for a few samples
Hada, T	2021	10.1186/s13048-021-00783-3	49	Carcinoma: 49, 25 infiltrative, 24 expansile invasion	CK7<br>CK20<br>CDX2<br>HER2<br>CK5/6<br>AR<br>ALDH1<br>CD24<br>CD133<br>PD-1<br>PD-L1<br>ZEB1<br>c-Met<br>EGFR<br>Snail-2	Various cancer associated proteins	Not reported	CK7 48/49<br>CK20 26/49<br>CDX2 44/49<br>HER2 17/49<br>CK5/6 10/49<br>AR 2/49<br>ALDH1 19/49<br>CD24 6/49<br>CD133 13/49<br>PD-1 2/49<br>PD-L1 12/49<br>ZEB1 2/49<br>c-Met 10/49<br>EGFR 7/49<br>Snail-2 2/49	EGFR, CD24 and CK5/6 enriched in infiltrative subtype and associated with poor survival
Hu, A	2015	10.1136/jclinpath-2015-202951	47	Borderline: 23<br>Carcinoma: 24	PAX8	Role in oncogenesis of the Mullerian system	Not reported	Borderline: 14/23<br>MOC: 11/24	Nuclear staining in more than 5% of malignant cells was considered positive. For positive cases, staining intensity was scored between 1-3.
Hunter, S	2012	10.1158/1078-0432.CCR-12-1103	95	Borderline	p16<br>p53<br>p-ERK<br>CK7<br>CK20	Various	Not reported	p16: 35%<br>p-ERK: 59%<br>p53: 21%<br>CK7: 98%<br>CK20: 57% focal; 21% diffuse	Staining intensity (none = 0, weak = 1, moderate = 2, strong = 3) and percentage of cells stained (0 = 0%, 1 = <1%, 2 = 1% to 10% ,3 = 10% to 33%, 4 = 33% to 66%, 5 = >60%). These scores were added for a final score of 0 to 8 for all stains.<br>NOTE: does not provide IHC scores for MOC.
Kang, E	2020	/10.1038/s41379-020-0618-9	113	Borderline: 12<br>Carcinoma: 101	TP53	Implicated in the progression of MBOT to MOC.	Not reported	Total: 77/113 demonstrated abnormal staining	Investigated if TP53 IHC is an accurate proxy for TP53 mutation status in ovarian mucinous tumours
Koh, I	2019	10.21873/cgp.20151	7	MOC: 7<br>Well-differentiated: 3<br>Moderately-differentiated: 1<br>Poorly-differentiated: 3	CDX2<br>Reg IV	CDX2: resistance to anticancer drugs;.<br>REG4 is a target gene of CDX2; increases expression of Bcl-2 and leads to inhibition of apoptosis.	Not reported	Well- and moderately differentiated MOC: 100% both CDX2 and REG IV<br>Poorly-differentiated: 0% expression	Correlation between expression of CDX2 and Reg IV and the degree of differentiation of cancer cells in MOC.<br><br>Positive (+): >50% of tumor cells stained;<br>Well-differentiated: Ratio of solid growth part 0-5%; Moderately-differentiated: 5-50%; Poorly- differentiated: 50-100%..

Lee, O-K	2015	10.1007/s00428-014-1688-1	8	Carcinoma: 8	CEACAM6	Role in cell adhesion, invasion, and metastasis. Inhibition of N-domain interferes with cell-cell interactions.	Other tumour types and normal tissue. Variably expressed	MOC: 7/8 cases positive expression.	Not compared to normal ovarian tissue, present in other normal epithelia.<br>Positive case defined as 10% or more staining along the luminal cell border or in the cytoplasm of the whole tissue section.
Leng, R	2015	10.1007/s12032-014-0329-5	40	Carcinoma: 40	Rac1	Implicated in tumour EMT, but limited information in EOC.	Normal ovarian tissue (n=24): 4.2%	Low expression: 45%<br>High expression 55%<br>Benign (n=41) and Borderline (n=26) tumours significantly lower expression levels but histological subtype (serous vs mucinous) was not given	Staining intensity scale of 0–3: 0, absence; 1, weakly stained; 2, moderately stained; and 3, strongly stained. The percentage of positive tumor cells on scale of 0–3: 0, no cells; 1, 33% positive tumor cells; 2, 33–66% positive tumor cells; and 3, 66% positive tumor cells. Staining score, 0 to 9, was the product of intensity and percentage scores. Low = 1-4, high = 5-9, and absence = 0.
Mackenzie, R	2015	10.1186/s12885-015-1421-8	44	Borderline: 26<br>Carcinoma: 37	p53	TP53 – known tumour suppressor, mutation may impart genomic instability	Not reported	MBOT:<br>12/15 cases - 1<br>1/15 cases - 2<br>2/15 cases - 0<br>MOC<br>2/29 cases - 0<br>17/29 cases - 1<br>10/29 cases - 2	Scoring system<br>0: complete absence<br>1: up to 50% nuclear positivity<br>2: greater than 50% positivity<br>High core failure rate, 11/26 MBOT and 8/37 were not interpretable
Mehner, C	2015	10.18632/oncotarget.5927	16	Carcinoma: 16	SPINK1	Autocrine factor capable of promoting ovarian cancer cell proliferation and survival.	Not reported	Negative: 6<br>Weak: 1<br>Intermediate: 5<br>Strong: 4	A composite score for both intensity and extent of staining: negative, no staining; weak, 1-10% intermediate or 1-5% strong intensity; intermediate, 10-40% intermediate or 5-10% strong intensity; strong, >40% intermediate or >10% strong intensity.
Mohammed, R	2016	10.1097/PGP.0000000000000302	48	Cystadenoma: 15<br>Borderline: 13<br>Carcinoma: 20	HER-2	Known oncogene in breast and gastric carcinoma	Normal ovarian epithelium: 0%	Benign: 0%<br>Borderline: 31%<br>Carcinoma: 55%<br><br>Variable expression levels.	IHC Score: conducted according to guidelines.<br><br>Also assessed HER-2 gene amplification
Montavon, C	2019	10.1007/s00432-019-03037-4	43	Benign: 31<br>Borderline: 10<br>Carcinoma: 2	LATS	Tumour suppressor protein	Not reported.	MOC: 0.125 median, 0.338 IQR but only 2 cases. Two cases of MBOT demonstrated a score of 2.<br><br>Low expression in MOT generally.	Scoring:<br>Weighted average score (intensity: 0-3, 0-100% of LATS1/2 expression) by three scientists independently.
Mueller, J	2018	10.1016/j.ygyno.2018.05.008	24	Carcinoma: 24	CK7<br>CK20<br>PAX8<br>ER<br>PR<br>PTEN<br>ARID1A/B<br>AF250a<br>MSH6<br>PMS2	Various	Normal epithelium and stroma for PTEN, MSH6, PMS2 and ARID1A.	CK7: 23/24<br>CK20: 16/24<br>PAX8: 21/24<br>ER: 6/24<br>PR: 3/24<br>PTEN: lost in 1/24<br>ARID1A: lost in 3/24<br><br>Variable staining.	CK7/CK20: only cytoplasmic staining was considered positive<br>PAX8: only nuclear staining was considered positive<br>ER/PR: positive when >1% of tumour nuclei showed immunoreactivity.<br>Loss of MSH6, PMS2 and ARID1A expression defined as complete absence of protein expression in tumour cell nuclei.<br>PTEN: lost if tumour cells displayed no immunoreactivity or less than internal control.<br><br>Poor description of IHC scoring system.

Muller, P	2019	doi.org/10.1186/s13048-019-0498-0	11	Carcinoma: 11	Tomm34	High levels promote cancer cell growth. Component of the cellular chaperone system Involved in the transport of mitochondrial preproteins .	Other OC types. Highest in SET.	Median: 0.8<br>Q1: 0.5<br>Q3: 1.15<br>Low level expression.	IHC score based on four staining intensities: 0-3.
O'Shannessy, D	2013	10.1097/PGP.0b013e3182774562	10	Carcinoma: 10	FRA (folate receptor-alpha)	Restricted expression in normal tissue. Expression levels correlated with tumour stage. Growth Advantage to cells expressing this receptor	Normal fallopian tube and cortical inclusion cysts: uniform intense staining of yepithelium	FRA Positive: 8/10. However, M score (each intensity x %/6, score out of 50) was on average ~20 for MOC, suggesting either low intensity and/or low % of cells were positive for most cases (and lower than normal fallopian tube).	IHC Score: staining intensity from 0-3+. Percentage of cells staining at each intensity in the core was also determined.<br><br>A sample (TMA core) was considered positive for FRA expression if the percentage of the tumor cells positive for membranous staining was >5% at any intensity.
Okamoto, T	2011	10.1038/modpathol.2010.204	86	Cystadenoma: 28<br>Borderline: 28<br>Carcinoma Stage I: 24<br>Carcinoma Stage II: 6	DPEP1	A zinc-dependent metallopeptidase that is involved in glutathione metabolism.	Not reported.	Non-Cystadenoma: 1/58 cases positive expression.	Positive expression quantified as percentage of positive cells in each sample, where >25% positive cells was seen as positive.<br><br>NOTE: very low expression profile in MOC.
Ozer, H	2012	10.1186/1746-1596-7-124	22	Cystadenoma: 12<br>Borderline: 7<br>Carcinoma: 3	p53<br>p21<br>bax<br>bcl-2<br>c-kit<br>telomerase<br>metallothionein	Various	Not reported.	Data presented as median (min,max).<br><br>Staining score of p53 significantly higher in MOC versus benign tumour.<br>Staining of p21, bax, c-kit, telomerase not statistically significantly different.<br><br>Staining of bcl-2 was negative.<br><br>Staining of Metallothionein was significantly lower in benign versus borderline.	*Nuclear staining was considered as positive for p53, p21, and telomerase if more than 1% cells stained. (+), (++), (+++), (++++) scores were given when 1%-10%, 11%-25%, 25%-50%, and more than 50% cells showed positive staining, respectively. Inappropriate scoring system for p53.*<br><br>*Cytoplasmic staining was interpreted as positive for bcl-2.*<br>*Membranous and cytoplasmic staining was considered positive for bax and c-kit.*<br>*Cytoplasmic and/or membranous staining was considered positive for metallothionein.*<br>*IHC scoring for these:. Intensity of the staining was scored as follows; 0 (no staining), 1 (weak), 2 (moderate), and 3 (strong). Percentage of the positive*

									cells were scored as 0 (no staining), 1 (10 <%), 2 (10-50%), 3 (51-80%), and 4 (80%>). These values were multiplied, resulting in possible scores from 0 to 12.
Park, K	2011	10.3858/emm.2011.43.2.011	7	Borderline: 3<br>Carcinoma: 4	AGR2	AGR2 up-regulated genes involved in cell proliferation, invasion, and angiogenesis	4 normal ovarian tissue samples	AGR2: 100% expression in all cases of MOC.	Expression at a basal level in normal ovary surface epithelium (weak or no expression)<br>NOTE: IHC scoring was not explained. Instead, a binary label of positive or negative expression was documented.
Plewka, D	2014	10.5603/FHC.2014.0015	45	Cystadenoma: 15<br>Borderline: 15<br>Carcinoma: 15	IL-1b<br>IL-6<br>TNF-a<br>TGF-b<br>COX-2<br>iNOS<br>NF-kB	Various	Not reported	IL-1b: 31% and 86% increased expression in borderline and malignant compared to benign.<br>IL-6: no expression in borderline and malignant MOC.<br>TNFa: 53% and 98% increased expression in MBOT and MOC compared to benign.<br>TGF-b: 55% and 66% increased expression in MBOT and MOC compared to benign.<br>COX2: 58 and 87%<br>iNOS: 40% higher in carcinomas compared to benign and MBOT<br>NF-kB: similar expression between all types	NOTE: all investigated proteins, except for IL-6 and NFkB, were expressed higher in MOC compared to benign and MBOT. However, no discussion surrounding normal stromal expression.<br><br>NOTE: only analyses positively staining cells.<br>“In each positively stained cell, the intensity of staining was measured as the optical density of the reaction product, with the image analysis program NIS AR (Nikon). For each analysed area an average optical density was calculated. Three sections for every studied protein and every patient were analysed. In each section ten randomly selected fields were examined. Finally, the arithmetic mean and standard deviation were calculated.”
Szubert, S	2013	10.1007/s00432-013-1569-z	13	Cystadenoma: 5<br>Carcinoma: 8	EMMPRIN<br>VEGF<br>bFGF	EMMPRIN (extracellular matrix metalloproteinase induce): stimulates the secretion of VEGF (angiogenesis). bFGF also angiogenesis related.	Normal ovarian tissue: 21	Non-significant elevated expression of VEGF and EMMPRIN in EOC compared to benign and normal ovarian tissue.<br><br>Non-significant reduced expression of bFGF in EOC compared to benign and normal ovarian tissue	NOTE: EMMPRIN expression in EOC was directly correlated with VEGF expression, suggesting early and/or reactivated angiogenesis in ovarian cancer. Aggressive EOC characterised by high EMMPRIN and VEGF.
Tanaka, Y	2011	10.4161/cbt.11.1.13877	3	Carcinoma: 3	EGFR		Not reported	2/3 positive	Positive if >70% of cells stained
Tang, L	2010	10.1016/j.ejca.2009.10.003	25	Borderline: 10<br>Carcinoma: 15	Integrin, a2<br>Fibronectin 1<br>NHE1<br>Crk<br>WAVE 1<br>GIT1<br>CKIe	Various	Normal ovarian stromal tissue: 10.	Statistically significant increase in expression between normal and invasive MOC for all proteins assessed except for GIT1	Unbiased selection of proteins through a plasma screening experiment<br>Intensity score (3+, strong positive stain; 2+, moderate stain; 1+, weak stain; 0, no evidence of stain) and percentage of positive cells (3+, most of cells stained; 2+, half of cells stained; 1+, few cells stained; 0, no cells stained). Multiplied for composite score<br>NOTE: only documents significance of protein expression. No scoring system to validate/reproduce findings.

					B-Catenin<br>CtBP-2				
Umezaki, Y	2013	10.12892/ejgo2578.2015	60	Cystadenoma: 24<br>Borderline: 15<br>Carcinoma: 21	S100P	Tumour susceptibility to paclitaxel and carboplatin; molecular mechanisms have not yet been elucidated	Not reported	Benign: 19/24 pattern 1; 5/24 pattern 2<br>MBOT: 13/15 pattern 2; 2/15 pattern 3<br>MOC: 8/21 pattern 2; 13/21 pattern 3	Results classified into three staining patterns.<br>1 - expressed in most nuclei of mucinous epithelial cells<br>2 - sporadically expressed<br>3 - absent or rarely expressed<br><br>NOTE: useful marker to differentiate benign from borderline from carcinoma, but the reduced expression with malignancy potential is not useful as a theranostic.
Tkalia, I.G.	2014	NA - PMID: 24980769	25	Carcinoma: 25<br><br>Stage I-II: 10<br>Stage III-IV: 15	ER<br>PR<br>TR	Steroid hormones regulate the level of not only own receptors, but also modulate content of receptors of the other hormones.	Not reported.	ER: 88.0%<br>PR: 84.0%<br>TR: 60.0%<br><br>Similar expression profile regardless of stage.	Degree of steroid receptor expression has been taken in scores:<br>0 - no staining of nuclei of tumour cells<br>1 - poor staining of < 10%<br>2 - 11-50%<br>3 - 51-80%<br>4 - >81%<br><br>NOTE: > 10% of moderate and high level of staining =positive
Vitiazeva, V	2015	10.1371/journal.pone.0130197	13	Benign: 6<br>Carcinoma: 7	MUC5AC<br>MUC16	May relate to tumour formation and progression	Normal epithelial ovarian cells no expression	Proportion of samples deemed positive:<br>MUC5AC: 4/4 in benign; 4/6 in malignant<br>MUC16: 3/4 in benign; 4/6 in malignant<br>No data for 2 benign and 1 MOC	The proportion of positive tumor cells were scored from 0–3 (None = 0, <1/3 = 1, 1/3-2/3 = 2, >2/3 = 3). The intensity of staining was scored from 0–3 (None = 0, Weak = 1, Moderate = 2, Strong = 3)..Any staining called positive. Two of the MOC were mixed, likely seromucinous
Webb, J	2015	10.1016/j.ygyno.2016.03.008	30	MOC: 30<br>Stage I: 17<br>Stage II: 12<br>Stage III: 1	PD-L1	Negative regulator of T cells. Indicator and inhibitor of anti-tumour immune responses.	Not reported.	8/30 (26.7%) of MOC were PD-L1 positive	TMA cores were evaluated by two independent observers and scored as positive or negative for PD-L1 using a threshold of ≥ 1 positive cells
Ye, F	2012	10.1159/000338078	152	Benign: 58<br>MBOT: 50<br>MOC: 44	PCLP1	Down-regulation may play a role in junction disruption	Highly expressed in normal ovarian epithelial tissue	Expression reduces with malignancy	NOTE: this paper finds a negative correlation between an expressed protein and tumour progression. Not useful in the context of targets for theranostics.
Young, J	2011	10.1186/1757-2215-4-6	6	MOC: 6	hRad17	Involved in DNA damage recognition and repair and is associated with accumulation of p53	Normal ovarian tissue - n=6	Overexpressed in 100% MOC cases, 13% benign.<br><br>Statistically significant increase in expression with malignancy potential.	A weighted score by multiplying the score (0-3) for intensity and the score for percentage stained (0-4). For percentage 0 represented no staining, 1, <25% stained, 2, 25-50% stained, 3, 50-75% stained, and 4 > 75% staining positive. Scores ≥3 were considered positive and scores <3 were considered negative.<br>NOTE: no clear expression profile in normal ovarian tissue.
Zhang, J	2010	10.1136/jcp.2009.073593	150	Benign: 62<br>Borderline: 47<br>MOC: 41	Octamer-4	Maintaining pluripotency and germline potential of pluripotent embryonic stem cells.	Normal ovarian epithelium: negative in 17/40:	Weak expression across all subtypes (no significant difference)<br>Benign: 37/62 (30 cases 1+)<br>Borderline: 32/47 (23 cases 1+)<br>Adenocarcinoma: 26/41 (63.4%) (12 cases 1+)	Staining intensity scored between 0-3.<br>Percentage stained scored between 0-3.<br>Final score (0-6) was obtained by summing those scores.<br>0=negative; 1-2 = 1+; 3-4= 2+;5-6= 3+

							1+ in 15/40<br>2+ in 8/40		NOTE: Oct4 did not show significant correlation with the clinical parameters of patients with mucinous carcinoma.
Zhang, W	2016	10.1007/s13277-015-4419-6	44	MOC: 44	CEP55	Unclear role in ovarian cancer. Has been shown to initiate cellular invasion and EMT in other tumour types	No or weak expression in normal ovarian tissue	20/44: no or weak expression<br>24/44: moderate or strong expression	The proportion of tumor cells: 1 (<10 % positive), 2 (10–50% positive), 3 (50–75% positive), and 4 (>75% positive). Intensity: 0 (no staining), 1 (weak staining= light yellow), 2 (moderate staining=yellow brown), and 3 (strong staining = brown).<br><br>NOTE: staining index score of ≥6 was used to define high CEP55 expression, and a score of ≤4 indicated low CEP55 expression.
Zhang, Y	2016	-	14	MOC	Pinin	Relieves CtBP1-mediated repression of E-cadherin expression	Normal ovarian tissue: mean score 0.29<br>n=8	MOC: 5.18	Score = by multiplying the proportion of the stained epithelial area (from 0 for absence to 3 for more than 95% of the total epithelial area) with the intensity (from 0 for negative staining to 3 for strongly positive staining).<br>Mean score obtained.

A further literature search was conducted with improved search terms. Table 2 below displays the targets that were localised to the cellular membrane and that fulfilled inclusion criteria.

Table 2 Characteristics of included immunohistochemistry studies in mucinous ovarian tumours (MOT) – Search 2. Targets that were not localised to the cellular membrane or that did not fit inclusion criteria were omitted.

First Author	Year	DOI	Total MOC Cases	Protein of Interest	Localisation	Goal	Control Tissue Expression	MOC Expression	Notes
**Hou, R**	2017	10.3892/ijmm.2017.3009	30	MUC1	Cell membrane	Expression profile	-	25/30 (83.33%)	Expression was higher in advanced MOC.<br>MUC1 and Lewis(y) expression is correlated
**Chen, C-K**	2014	10.1097/MD.0000000000000171	49	HER2	Cell membrane	Expression profile	-	11/49 (22.44%)	Compared ASCO/CAP 2007 and 2013 staining techniques for concordance. Results taken from the 2013 scoring methodology, where staining score of 2+ was considered positive/elevated HER2 expression and rate of concordance with FISH was higher.
**Kobel, M**	2014	10.1038/bjc.2014.567	193	FOLR1	Cell membrane	Expression profile	-	3/193 (3.1%)	Samples were considered positive if there was strong membranous expression (50%) of FOLR1.
**Missaoui, N**	2014	10.7314/apjcp.2014.15.19.8121	14	HER2	Cell membrane	Expression profile	-	14.30%	Stained according to the 2007 ASCO/CAP guidelines
**Wang, J**	2014	10.1111/his.12578	19	MUC1	Cell membrane	Expression profile	-	31.60%	MUC2, MUC16, MUC5AC - low cancer specificity<br>6/19 moderately positive expression in 10-90%<br>6/19 weakly positive expression<br>7/19 no expression<br>No comment on tumour-associated MUC1 type
**Chao, WR**	2014	10.1097/PAS.0000000000000268	49	HER2	Cell membrane	Expression profile	-	9/49 (18.37%)	2013 ASCO/CAP guidelines<br>100% concordance with HER2 gene copy amplification
**Kim, SK**	2014	-	46	HER2	Cell membrane	Expression profile	-	37.84% (14/46)	2007 ASCO/CAP guidelines
**Yan, B**	2011	10.1136/jclinpath-2011-200082	17	HER2	Cell membrane	Expression profile	-	29.4% (5/17)	Relatively concordant with FISH analysis of gene amplification<br>Not specifically ASCO/CAP guidelines, but similar rules

**Van Elssen, CH**	2010	10.1111/j.1365-2559.2010.03667.x	5	MUC1	Cell membrane	Expression profile	-	4/5 (80%)	Assessed cancer-associated glycoform of MUC1<br>Differentiation-associated glycoform found in 6% (3/5) of MOC samples.

Table 3 Characteristics of included proteomics studies

First Author	Year	DOI	Total MOC Cases	Identification Technique	Validation Technique	Protein of Interest	Subcellular Localisation	Role of Target	Control	Findings	Notes
Tian, Y	2011	10.1002/pmic.2011	3	LC-MS/MS Quantification	Western Blotting	CEA5;<br>CEA6;<br>MUC1;<br>MUC2;<br>MUC5b;<br>ACE2;<br>60 kDa heat shock protein, mitochondrial;<br>Fc fragment of IgG binding protein FCGBP;<br>GP2;<br>PTPRN2	CEA5: cell surface;<br>CEA6: cell surface;<br>MUC1: cell surface;<br>MUC2: extracellular region;<br>MUC5b: extracellular region;<br>ACE2: plasma membrane and extracellular region;<br>60 kDa heat shock protein, mitochondrial: mitochondrial;<br>Fc fragment of IgG binding protein FCGBP: extracellular region;<br>GP2: plasma membrane;<br>PTPRN2; membrane<br>Protein tyrosine phosphatase	Various	Human International Protein Index<br><br>NCBI Reference Sequence	CEA5/6 quantified through WB	Proteins with at least a twofold change were considered to have altered expression.
Longuespee,	2012	10.1007/s00418-953-0	25	MALDI TOF MS	IHC	C-terminal fragment of the 11S proteasome activator (PA28, Reg alpha)	Benign: nuclear<br>Malignant: cytoplasmic	MHC-class I antigen processing	Swiss-Prot database filtered for the taxonomy "human"	Significant proteomic expression compared to control	From 2007 publication: Only protein sequences with MOWSE score higher than 32 (indicating significant homology or identity) and identified in several samples representing 3 significant MS/MS were considered.
Toyoma, A	2012	10.1111/j.1349-2012.02224.x	6	MALDI TOF MS	Western Blotting	Serpin B5	extracellular space	Known TSG in breast cancer and oncogene in other cancers.	20239 human sequences of UniProt	4.76 fold chance<br>p-value: 1.0E04	Confounded by unmatched sample size.
