# Supplementary material for: The Protein Landscape of Mucinous Ovarian Cancer: Towards a Theranostic
Source: Cancers (Basel). 2021 Nov 9;13(22):5596. doi: 10.3390/cancers13225596 (PMC8616050; doi:10.3390/cancers13225596)
Supplement: Supplementary file 1 [file cancers-13-05596-s001.zip › Supplementary File S3 - Target Characteristics[1].pdf]

Supplementary File S3: TARGET CHARACTERISTICS

Table 1 Characteristics of proteins in immunohistochemistry studies

Protein[1]	Number of Included Studies	Expression in MOC compared to controls/other tissues[2]	Cellular localisation[3]
AGR2	2	Elevated	C
AMACR	1	Elevated	C
ARID1A	1	Non-specific	N
B-CATENIN	1	Non-specific	M
BAX	1	Non-specific	C, M
BCL2	1	Non-specific	C, M
C-KIT	1	Reduced	NA
MUC16	1	Non-specific	M, S
CA19.9	1	Non-specific	G
CDX2	5	Non-specific	N
CEACAM6	1	Elevated	CS, CM
CEP55	1	Elevated	C
CHEK1	1	Non-specific	N
CK20	5	Non-specific	C
CK7	5	Non-specific	C
CKIe	1	Elevated	C, N
COL3A1	1	Elevated	C
COX2	1	Non-specific	N
CRK	1	Elevated	C, N
CtBP-2	1	Elevated	N
DPEP1	1	Reduced	NA
EMMPRIN	1	Reduced	NA
ER	2	Elevated	N
FGF18	1	Elevated	C
FIBRONECTIN 1	1	Elevated	S
FOXM1	1	Elevated	C
FRA	2	Elevated	S, CM, E
GIT1	1	Reduced	NA
GPR158	1	Elevated	CM
HER2	4	Elevated	CM, N, E, C
HNFIB	1	Non-specific	N
hRad17	1	Elevated	N
IL-1B	1	Non-specific	NA
IL-6	1	Non-specific	NA
INI	1	Non-specific	N
INOS	1	Non-specific	C
INTEGRIN, A2	1	Non-specific	M
KIF23	1	Non-specific	N
LATSR	1	Reduced	NA

MDM2	1	Elevated	N
METALLOTHIONEIN	1	Elevated	C
MTAP	1	Non-specific	C, N
MUC2	2	Non-specific	S
MUC5AC	2	Non-specific	S
NF-KB	1	Non-specific	N
NHE1	1	Elevated	CM, ER
Octamer-4	1	Reduced	NA
P16	1	Elevated	N
P21	1	Elevated	N
P53	6	Elevated	N
PARPBP	1	Elevated	N
PAX2	2	Elevated	N
PAX8	6	Elevated	N
PCLP1	1	Reduced	NA
PD-L1	1	Elevated	CM
pERK1/2	2	Elevated	N
Pinin	1	Non-specific	N
PR	2	Reduced	NA
PTEN	1	Reduced	NA
RAC1	1	Elevated	CM
REG4	2	Elevated	S
S100B	1	Reduced	NA
SPINK1	1	Elevated	S
TELOMERASE	1	Reduced	NA
TFF3	1	Elevated	C, S
TGF-B	1	Elevated	N
TNF-A	1	Elevated	N
TOMM34	1	Reduced	NA
TR	1	Elevated	N
WAVE1	1	Elevated	C

1. Name as reported in the original study.
2. Non-specific: has high or ubiquitous expression in other normal tissues; Reduced: reduced or insignificant expression in MOC compared to ovarian stromal tissue; Elevated: higher expression in MOC than control or normal tissues.
3. NA, not assessed; CS, cell surface; CM, cell membrane; E, endosome; C, cytoplasmic; N, nuclear; S, secreted; M, mitochondrial; G, Golgi Apparatus; ER, endoplasmic reticulum.

A further literature search was conducted with improved search terms. Table 2 below displays the targets that were localised to the cellular membrane and that fulfilled inclusion criteria.

Table 2 Characteristics of included proteins using the revised search strategy.

Protein[1]	Number of Included Studies	Expression in MOC compared to controls/other tissues[2]	Cellular localisation[3]
CEACAM5	1	Elevated	CM
CEACAM6	1	Elevated	CM
EGFR	4	Elevated	S, CM, N, E
FOLR1	1	Elevated	CM
HER2	5	Elevated	CM
MUC1	3	Elevated	CM

1. Name as reported in the original study.
2. Non-specific: has high or ubiquitous expression in other normal tissues; Reduced: reduced or insignificant expression in MOC compared to ovarian stromal tissue; Elevated: higher expression in MOC than control or normal tissues.
3. NA, not assessed; CS, cell surface; CM, cell membrane; E, endosome; C, cytoplasmic; N, nuclear; S, secreted; M, mitochondrial; G, Golgi Apparatus; ER, endoplasmic reticulum.

Table 3 Characteristics of proteins in proteomic studies

Protein[1]	Expression in MOC compared to controls/other tissues[2]	Cellular Localisation[3]
CEA5 (CEACAM5)	Elevated	CM, CS
CEA6 (CEACAM6)	Elevated	CM, CS
MUC1	Elevated	CM
MUC2	Elevated	S
MUC5B	Elevated	S
ACE-like protein (ACE2)	Elevated	S, CM, C
60 kDa heat shock protein, mitochondrial	Elevated	M
Fc fragment of IgG binding protein FCGBP	Elevated	S
Pancreatic secretory granule membrane major glycoprotein GP2 (GP2)	Elevated	S, CM
Protein tyrosine phosphatase (PTPRN2)	Elevated	CM, C
C-terminal fragment of the 11S proteasome activator (PA28, Reg alpha)	Elevated	C, N
Serpin B5	Elevated	S

1. Name as reported in the original study, with the common name in brackets.
2. Non-specific: has high or ubiquitous expression in other normal tissues; Reduced: reduced or insignificant expression in MOC compared to ovarian stromal tissue; Elevated: higher expression in MOC than control or normal tissues.
3. NA, not assessed; CS, cell surface; CM, cell membrane; E, endosome; C, cytoplasmic; N, nuclear; S, secreted; M, mitochondrial; G, Golgi Apparatus; ER, endoplasmic reticulum.
